# The Paradox of Rising Ethnic Prejudice in Times of Educational Expansion and Secularization in the Netherlands, 1985–2011

**DOI:** 10.1007/s11205-017-1718-x

**Published:** 2017-08-01

**Authors:** Paula Thijs, Manfred Te Grotenhuis, Peer Scheepers

**Affiliations:** Department of Sociology, Radboud University, P.O. Box 9104, 6500 HE Nijmegen, The Netherlands

**Keywords:** Ethnic prejudice, Longitudinal change, Educational expansion, Secularization, Counterfactual analysis

## Abstract

We aim to clarify a puzzling paradox: while shares of highly educated and non-religious individuals—who generally hold less prejudice—have increased in the Netherlands, levels of prejudice against ethnic minorities have yet risen over time. To solve the paradox, we use cross-sectional data from 1985 to 2011 in counterfactual analyses. In these analyses we simulate that levels of ethnic prejudice within categories of education, church membership, and church attendance are kept constant at the 1985 level and a new simulated trend in prejudice is estimated for the 1985–2011 period. Our findings show that changing levels of prejudice within categories of education are partly responsible for the trend. We conclude that the increasing share of highly educated individuals has not resulted in a decline of prejudice in the Netherlands over time, because all Dutch have become more prejudiced over the years and in particular the higher educated.

## Introduction

Throughout the twentieth century, Western European societies have witnessed a considerable increase in educational levels (Meyer et al. 1977; Schofer and Meyer 2005). Over the same period, religious affiliation and religious participation have declined (Norris and Inglehart 2011). The Netherlands is a leading country in educational expansion and secularization (Bar Haim and Shavit 2013; Becker and De Hart 2006). Since the second half of the twentieth century, the educational level of the Dutch has risen substantially (Tolsma and Wolbers 2014; Van Hek et al. 2015), which coincided with a strong decline in religious affiliation, church attendance, and traditional Christian beliefs (De Graaf and Te Grotenhuis 2008; Need and De Graaf 1996).

Previous research consistently found higher educated individuals to show less ethnic prejudice compared to lower educated (Coenders and Scheepers 2003; Hello et al. 2002; Wagner and Zick 1995). Also non-religiously affiliated people and people who do not attend church were found less prejudiced than church members and regular churchgoers (Allport and Ross 1967; Scheepers and Eisinga 2015). Thus, education, religious affiliation, and attendance are statistically related to ethnic prejudice at the individual level, while the shares of highly educated and non-religious individuals have increased. Consequently, one would expect a longitudinal decline in prejudice at the national level.

In contrast, levels of ethnic prejudice seem to have risen in the Netherlands. Several scholars found more widespread support for ethnic discrimination in the housing and labor markets since the late 1980s (Coenders et al. 2008; Coenders and Scheepers 1998; Huijnk and Dagevos 2012), and stronger support for ethnic prejudice since the 1990s (Coenders et al. 2015). Similar changes have been found in other European countries (see Ceobanu and Escandell (2010) for an overview). For example, Semyonov et al. (2006) found a rise in anti-foreigner sentiment between 1988 and 2000 in 12 European countries. Other studies showed considerable differences between European countries in the direction and magnitude of changes in anti-immigrant and anti-immigration attitudes since 2000, with attitudes recently becoming somewhat less negative in several countries, including among others the Netherlands, Germany and Poland (Hjerm and Bohman 2014; Meuleman et al. 2009; van Setten et al. 2017). Although there are indications that Dutch public opinions on ethnic diversity have recently become milder (Huijnk and Andriessen 2016), the documented rise in ethnic prejudice over the past decades gives rise to a puzzling paradox: while the shares of higher educated people and non-affiliated people in Dutch society—known for their relatively lower levels of prejudice—have increased over time, prejudice against ethnic out-groups has yet increased.

There are two possible explanations for this. First, ethnic prejudice may have risen to such a degree throughout society that it has offset the impact of educational expansion and secularization. In this scenario, the levels of ethic prejudice within *all* categories of education and religious affiliation have risen to the same extent. Second, the levels of ethnic prejudice may have risen particularly within the higher educated and the non-affiliated. As a consequence, educational expansion and secularization could then not have reduced prejudice in society.

Few studies, however, have examined whether the relationship of education and religious affiliation with prejudice has changed over time and the results are mixed. For example, Quillian (1996) and Jaspers (2008) showed that the effect of education increased over time in the U.S. and in the Netherlands, whereas Easterbrook et al. (2016) found stable relationships between education and anti-immigrant attitudes over time based on British and international surveys. In their meta-analysis, Hall et al. (2010) found decreasing associations between extrinsic religiosity and ethnic prejudice over time, while Jaspers (2008) found no changes in the effect of church membership over time in the Netherlands.

In addition, if the relationship of education and religious affiliation with ethnic prejudice has indeed changed, it remains unclear *which* educational and religiously (non-)affiliated groups are responsible for these changes by expressing more or less ethnic prejudice over time. In a Dutch study, de Lange et al. (2015) found a widening gap in ethnic threat between higher and lower educated, caused by a stronger increase among the latter. However, the authors only considered linear trends. We therefore examine whether the levels of ethic prejudice within categories of education, church membership, and church attendance have changed over time at any (non-linear) rate.

Most importantly, it remains unknown to what extent these changes in the levels of ethic prejudice can explain the longitudinal rise in ethnic prejudice *despite* educational expansion and secularization. Although few studies considered the effect of changing levels of prejudice within particular groups, none of these studies took distributional shifts of these groups into account, which may have resulted in biased effects. To overcome this, we use counterfactual analyses (Te Grotenhuis et al. 2004). We examine whether the rise in ethnic prejudice is still present if the levels of ethic prejudice within categories of education and religious affiliation would not have changed over time, while controlling for distributional shifts, i.e., educational expansion and secularization. This leads to the following research questions:

- Have the higher educated and the non-affiliated become more prejudiced than others between 1985 and 2011?
- If so, to what extent have these changes contributed to the observed rise in prejudice in the Netherlands between 1985 and 2011, while taking into account educational expansion and secularization?

Rising levels of ethnic prejudice may have detrimental consequences for interethnic relations in society. Ethnic prejudice may lead to negative intergroup behavior, such as discrimination, exclusion or hostility (Allport 1954). Feelings of exclusion and discrimination may hamper ethnic minorities’ social integration and increase withdrawal into one’s own ethnic group, which could eventually result in radicalization or criminalization (Huijnk and Dagevos 2012). Understanding to what extent the rise in ethnic prejudice can be explained by certain groups in society expressing more prejudice over time may inform policies to reduce interethnic tensions.

## Theories and Hypotheses

### Ethnic Competition Theory

On the individual level, ethnic competition theory has proven fruitful to explain differences in ethnic prejudice between the higher and lower educated (Coenders and Scheepers 2003; Hello et al. 2002; Wagner and Zick 1995), and between non-religious and religiously affiliated individuals (Allport and Ross 1967; Scheepers and Eisinga 2015; Scheepers et al. 2002). Competition between in-group members and ethnic out-group members over scarce economic or cultural resources poses a real threat to the social position of the in-group as a whole, and in particular to those competing more severely with ethnic out-groups (Blalock 1967; Coenders 2001). This encourages perceptions of interethnic threat, which in turn induce ethnic prejudice and exclusionism (Quillian 1995; Scheepers et al. 2002).

In general, ethnic minorities have more disadvantaged socio-economic positions and less education compared to the average population (Gijsberts et al. 2012). Natives with lower education are more likely to hold similar economic positions to ethnic minorities than higher educated natives. Lower educated natives may therefore have stronger perceptions of threat from ethnic minorities over *economic resources*, such as jobs and social security benefits than higher educated individuals, which induces prejudice against ethnic minorities (Hello et al. 2002, 2006). Higher educated individuals may perceive less ethnic threat because they compete less with ethnic minorities, but also because they are less susceptible to ethnic threat. It is argued that education increases awareness to alternative viewpoints and broadens people’s perspectives, including ideas of cultural relativity and diversity (Gabennesch 1972). As a consequence, higher educated individuals will be better able to recognize cultural expressions and more willing to accept cultural and ethnic differences (Manevska and Achterberg 2013). Likewise, the educational system is argued to transmit democratic norms and values that emphasize individual and cultural freedom and enables pupils to generalize these principles to minority groups (Vogt 1997). The higher people’s educational level, the longer their exposure to this ‘liberalizing’ influence of education and the less ethnic prejudice they have (Hello et al. 2002).

Competition between Dutch natives and ethnic minorities may also concern *cultural resources*, that is, belief systems and dominant cultural norms and values. Ethnic minorities often have different cultural norms and values and belong to non-Christian religions, or are perceived as such by Dutch natives. Consequently, these conflicting values may be perceived as a threat to the central values of the in-group (Schneider 2008; Stephan and Stephan 2000), which could evoke prejudice against any ethnic out-group (Sniderman et al. 2004). Religiously affiliated natives may perceive ethnic minorities’ beliefs as a threat to their own religious beliefs and practices, which induces higher levels of prejudice among the religiously affiliated than among non-affiliated people (Coenders 2001; McLaren 2003).

### Changing Levels of Ethnic Prejudice: Education

According to ethnic competition theory, actual economic competition increases perceptions of ethnic threat, in particular among those who are in similar socio-economic positions as most members of ethnic out-groups. During the second half of the twentieth century, the number of low educated, unskilled migrants has increased in the Netherlands and the established minority groups maintained a disadvantaged socio-economic position compared to Dutch natives (Gijsberts et al. 2012; Gijsberts and Lubbers 2013). Consequently, ethnic competition has increased among lower educated individuals in particular, which could have induced higher levels of ethnic prejudice among the lower educated.

In addition, modernization has considerably improved educational opportunities for children from lower socio-economic backgrounds (Breen and Jonsson 2005). As a result, the group of lowest educated people in Dutch society has become smaller and more homogeneous with fewer cognitive, financial, and social resources (Gesthuizen et al. 2005), and less cultural capital (Manevska and Achterberg 2013). Following ‘the losers of modernization’ thesis, the lowest educated categories in society lack sufficient cultural capital to get ahead in a rapidly changing world and find themselves in an increasingly vulnerable and isolated social position (Betz 1994). As a result, lower educated individuals may have become more susceptible to perceptions of ethnic threat, inducing higher levels of ethnic prejudice among these individuals. Both perspectives predict that the average level of prejudice has risen because the lower educated—who already held more ethnic prejudice—have become even more prejudiced than before. We hypothesize that *particularly lower educated people have become more prejudiced over time, which consequently increased the general level of ethnic prejudice in the Netherlands* (H1a).

Although the established minority groups in the Netherlands still have a disadvantaged socio-economic position compared to the native majority, their educational level and participation in senior or academic level occupations have improved over the past decades (Dagevos and Gijsberts 2010; Gijsberts 2004). Following ethnic competition theory, middle and higher educated natives may therefore have increasingly perceived economic threat from ethnic out-groups, inducing higher levels of ethnic prejudice among these categories. Lancee and Sarrasin (2015), for example, found that higher educated individuals in Switzerland show more negative attitudes towards immigrants once they enter the labor market on which they compete with ethnic minorities.

Moreover, increased educational opportunities due to modernization have likely resulted in a more heterogeneous group of higher educated individuals, with more variation in parental background, cognitive abilities, and cultural capital. Although scholars have demonstrated the importance of the educational system on reducing people’s ethnic prejudice, others also found that part of the educational effect originates from differences in cultural capital (Manevska and Achterberg 2013), or even from factors that influence people’s level of ethnic prejudice before they attend secondary education (Lancee and Sarrasin 2015), such as authoritarianism (Hello et al. 2006). The heterogenization of higher education may therefore imply that people who are more susceptible to ethnic threat have increasingly attained higher educational levels. As a result, higher educated individuals as a group may have become more prejudiced over time, thereby reducing the liberalizing effect of educational expansion. Based on these arguments, we expect that *the higher educated have converged towards the already prejudiced lower educated over time, which consequently increased the general level of ethnic prejudice in the Netherlands* (H1b).

### Changing Levels of Ethnic Prejudice: Church Membership and Attendance

Following ethnic competition theory, strong identification with a religious group, as well as the belief that one’s religion is the only true religion, increase (perceptions of) competition with out-group members from different religions (Ekici and Yucel 2014; Glock and Stark 1965; Scheepers et al. 2002). Due to immigration of in particular non-Christian migrants, cultural and religious diversity have increased considerably in the Netherlands. The percentage Muslims as part of the Dutch population has risen from 0.4% in 1971 (Statistics Netherlands 2004) to 5% in 2012 (Maliepaard and Gijsberts 2012). Hence, over time, Christian natives have likely perceived increasing threat from ethnic minorities belonging to other religions, especially in a secularizing country as the Netherlands (Becker and De Hart 2006; McLaren 2003). As a consequence, people affiliated to Christian churches may have become more inclined to preserve their religious identity by stressing the boundaries between the Christian religious in-group and other (non-Christian) out-groups, increasing their levels of ethnic prejudice. Based on these arguments, church members and regular churchgoers may have become even more prejudiced over time. Thus, we expect that *particularly church members* (H2a) *and regular churchgoers* (H2b) *have become more prejudiced over time, which consequently increased the general level of ethnic prejudice in the Netherlands*.

On the contrary, the increased salience of cultural and religious threat posed by ethnic minorities may have also affected the non-affiliated part of the Dutch population. Due to modernization and individualization, the significance of traditional Christian norms and values has diminished over the past decades (Felling et al. 2000; Inglehart 1997). At the same time, the increasingly secular Dutch population is confronted with rising numbers of ethnic minorities belonging to non-Christian religions (Maliepaard and Gijsberts 2012). Hence, the debate on ethnic minorities has become centered around value conflicts. Ethnic minorities have often been framed as undermining liberal and democratic ‘Dutch’ values, such as gender equality and tolerance towards homosexuals, which are particularly cherished by the secular part of the population (Koopmans 2015; Vasta 2007). It is argued that secular natives want to defend their liberal values against the perceived moral conservatism of ethnic minorities’ religions (Schuh et al. 2012). This could have induced higher levels of prejudice among the non-affiliated in particular, thereby reducing the positive effect of secularization. We therefore hypothesize that *non*-*church members* (H2c) *and non*-*churchgoers* (H2d) *have converged towards the already prejudiced religiously affiliated over time, which consequently increased the general level of ethnic prejudice in the Netherlands*.

## Data and Methods

### Data

To test our hypotheses, we used data from the Socio-cultural Developments in the Netherlands (SOCON) surveys. Between 1979 and 2011, seven cross-sectional waves were conducted, with each successive wave being a replication and extension of the previous waves (Eisinga et al. 2012). The methodological design of the repeated cross-sections has remained largely similar and comparable over time. Each survey consists of a representative sample of the Dutch population between 18 and 70 years, and contains questions on a wide variety of social issues derived from previously tested, valid, and reliable measurements. We used the surveys of 1985, 1995, 2000, 2005, and 2011, which were combined into one pooled data-set. The surveys of 1979 and 1990 were excluded because comparable measures on ethnic prejudice were missing. Questions measuring ethnic prejudice were administered solely to respondents whose nationality and that of their parents and grandparents. Therefore, our study only includes native Dutch individuals.

### Measurements

#### Dependent Variable

To measure ethnic prejudice, respondents were presented five statements indicating prejudice against ethnic out-groups: ‘With Moroccans you never know for certain whether they are going to be aggressive or not’, ‘Most people from Surinam work quite slowly’, ‘Gypsies are never to be trusted’, ‘Turks are backward’ and ‘When you do business with Jews, you have to be extra careful’ (Cronbach’s alpha = 0.77, see Table 5 in “Appendix 1”). These statements are based on common stereotypes about Moroccans, Turks, and Surinamese, which are members of the three largest out-groups in the Netherlands, and gypsies and Jews, which had often been the object of prejudice and derogation in the past (Hagendoorn and Janssen 1983). The items are a selection of a wider range of similar items which were previously tested and often used to measure prejudice against ethnic minorities (Scheepers et al. 1990). Response categories on each statement ranged from (1) ‘agree entirely’ to (5) ‘do not agree at all’, constituting a five-point Likert-scale. The response category ‘never thought about’ was excluded from analysis.

In 1985 and 1995, the five questions on ethnic prejudice were filled out by a random subsample of the total sample in these waves, resulting in a total sample size of 5530 respondents. To further limit the loss of cases on the dependent variable due to missing answers (1256 respondents), we conducted multiple imputation of missing values in SPSS for respondents with a valid answer on at least three of the five items. Respondents with missing answers on more than two of the five items were excluded from the analyses. Five sets of imputed values were independently drawn, which were combined into one pooled set on which the analyses were performed. After imputation, 5229 respondents with a valid answer were left.

Factor analysis of the five items indicated that the items refer to one single underlying dimension.^1^ Separate factor analyses per wave showed acceptable factor loadings and communalities, which were comparable across the surveys (see Table 5 in “Appendix 1”). Factor scores were computed, representing the weighted linear combination of the five items.^2^ We subtracted the minimum value from the factor scores to have the dependent variable starting at zero. A higher score on the scale indicates more ethnic prejudice.

#### Independent Variables

*Educational attainment* was measured as the respondent’s highest education completed after elementary school. Response categories were recoded into seven categories ranging from primary education to master’s or equivalent level and higher. Responses on the ‘other’ category were treated as missing values (0.2%) and excluded from analysis. Between 1985 and 2011, the relative share of lower educated individuals decreased significantly from 14.3 to 2.8% for people with only primary education, while the relative share of highly educated individuals increased significantly from 3.5 to 11.4% for people holding a master’s degree or equivalent (see Fig. 4 in “Appendix 2”).

To measure religious affiliation, we used both church membership and church attendance. Respondents were first asked whether they considered themselves a member of a (Christian) church or religious community (yes/no). Next, respondents who answered ‘yes’ were asked which church or religious community they considered themselves a member of. We combined these questions into one variable indicating *church membership* and reduced the response categories to Catholic, Protestant, other Christian, non-Christian, and non-member. We excluded individuals belonging to other Christian and non-Christian religions because of their marginal numbers in the sample (<5%), and because secularization mainly concerns the Catholic and Protestant churches in the Netherlands (Becker and De Hart 2006). *Church attendance* was measured by a question on the frequency at which one attends services of a church or religious community: about once a week, about once a month, once or twice a year or hardly ever/never. Respondents who had no valid answer on the variable for church attendance (0.2%) were excluded from analysis. Between 1985 and 2011, the relative share of people considering themselves non-religious increased significantly from 47.3 to 68.3%. The percentage hardly ever or never attending services of a church or religious community increased significantly from 43.1% in 1985 to 58.3% in 2011 (see Figs. 5, 6 in “Appendix 2”).

### Control Variables

Birth cohort, sex, socio-economic position, degree of urbanization, and province were included as control variables in the analyses, as these characteristics are shown to be related to the dependent variable (Coenders and Scheepers 1998), and could possibly confound the relation of education, church membership, and church attendance with prejudice.^3^ *Birth cohort* was derived from the respondent’s birth year. A linearity test indicated that we could include birth cohort linearly in the analyses without significant loss of explanatory power. *Sex* was measured as male (0) or female (1). To obtain one measure for *socio*-*economic position*, the EGP classification of social class (Erikson et al. 1983) and main activity of the respondent were combined. *Degree of urbanization* was based on a measure of address density per square kilometer provided by Statistics Netherlands (den Dulk et al. 1992), divided in five categories.^4^ *Province* was measured as which of the twelve Dutch provinces the respondent was living in at the time of the interview. To control for possible non-linear relations between the dependent variable and the independent variables, dummy variables were created for each category of our individual and control characteristics except for birth cohort. Missing values on the independent and control variables were deleted listwise, resulting in a sample size of 4780 respondents in our analyses. See Table 1 for descriptive statistics for all variables in the analysis.

**Table 1:** Descriptive statistics of dependent and independent characteristics (N = 4780). *Source*: SOCON 1985–2011 (Eisinga et al. 1992, 1999, 2012)

	Percentage	Mean	SD	Min	Max
Ethnic prejudice		1.74	0.88	0.00	4.52
*Educational attainment*					
Primary education	8.7				
Lower vocational education	16.5				
Lower secondary vocational education	13.7				
Secondary vocational education	22.6				
Upper secondary education	12.1				
Bachelor’s or equivalent level	18.8				
Master’s or equivalent level	7.6				
*Church membership*					
Catholic	23.7				
Protestant	17.9				
Non-religious	58.5				
*Church attendance*					
Yes, about once a week	14.4				
Yes, about once a month	8.5				
Yes, once or twice a year	25.7				
No, hardly ever/never	51.5				
Cohort		1954.04	15.68	1915	1993
*Sex*					
Male	49.0				
Female	51.0				
*Socio*-*economic position*					
Professional	26.2				
Other white collar	17.6				
Self-employed	3.6				
Skilled manual	5.7				
Unskilled manual	9.6				
Unemployed	6.4				
Retired	8.7				
Housekeeping	14.6				
Education	5.0				
Other	2.6				
*Urbanization*					
Very strong urbanization	17.6				
Strong urbanization	23.4				
Moderate urbanization	20.1				
Little urbanization	20.1				
No urbanization	18.7				
*Province*					
Groningen	3.4				
Friesland	5.0				
Drenthe	3.7				
Overijssel	6.9				
Gelderland	13.3				
Utrecht	6.8				
Noord-Holland	13.9				
Zuid-Holland	21.0				
Zeeland	3.6				
Noord-Brabant	13.4				
Limburg	9.0				

### Method

To investigate whether and how prejudice has changed over time within the different categories of educational attainment, church membership, and church attendance, we used multivariate OLS regression models. To account for non-linear changes, we performed these analyses for each survey year separately. Next, we calculated mean levels of prejudice for each category of educational attainment, church membership, and church attendance, while controlling for all relevant variables. Secondly, we examined the relative contribution of these changes to the observed trend in prejudice in a counterfactual simulation analysis (Te Grotenhuis et al. 2004). We simulated the trend in prejudice as if the differential changes in prejudice within each category of education, church membership, and attendance had not taken place since 1985. We chose 1985 as a reference point, because educational expansion and secularization were on their lowest in the period under study. We used the predicted levels of prejudice in 1985 within all categories of education, church membership, and church attendance as found in the multivariate regression models to estimate a new (simulated) mean level of prejudice in each subsequent survey year, while taking into account the rising shares of higher educated and non-religious individuals. The differences between the observed and the simulated trends in prejudice show to what extent the observed trend is due to differential changes in prejudice within categories of education, church membership, and church attendance between 1985 and 2011. See “Appendix 3” for an explanation of this counterfactual simulation method using a straightforward bivariate example for two survey years.

## Results

### General and Differential Trends in Prejudice

**Fig. 1:**
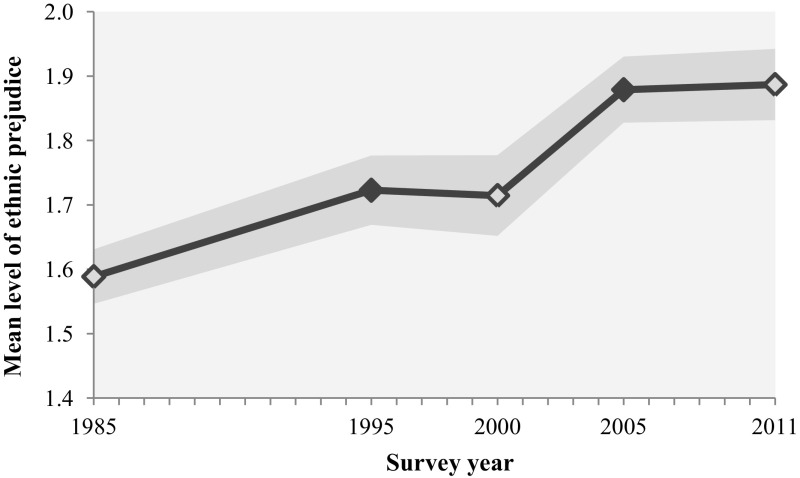
The trend in ethnic prejudice in the Netherlands, 1985–2011 (N = 4780). *Note filled marker* indicates significant mean difference compared to previous survey year. *Grey area* shows the 95% confidence interval. *Source*: SOCON 1985–2011 (Eisinga et al. 1992, 1999, 2012)

Figure 1 shows the general trend in ethnic prejudice over time. We observe a significant increase in prejudice against ethnic minorities (Moroccans, Turks, Surinamese, gypsies, and Jews) from 1.16 in 1985 to 1.89 in 2011 (on the scale ranging between 0 and 4.52).^5^ Between 1995 and 2000, and between 2005 and 2011 the change in ethnic prejudice was not significant.^6^

Table 2 shows the associations of education, church membership, and church attendance with ethnic prejudice per survey year, based on multivariate OLS regression analyses. For ease of interpretation we transformed the regression coefficients such that the intercept represents the weighted (sample) mean level of prejudice in each year, while the dummy variable coefficients reflect the deviance from this overall sample mean (Sweeney and Ulveling 1972; Te Grotenhuis et al. 2016). In line with previous research, Table 2 shows a positive relationship of educational attainment with prejudice. In each survey year, lower educated individuals held significantly more prejudice than average, while the higher educated (upper secondary education or higher) held significantly less prejudice than average. In 1985, for example, the mean level of prejudice among people with primary education lies 0.38 points above the average of 1.59. People with master’s or equivalent level show a prejudice level that lies 0.60 points below the average of 1.59. The standardized coefficient (beta) shows a considerable impact of education, which seems to have slightly decreased over time. The influence of church membership and church attendance on prejudice was very small, as indicated by the low standardized coefficients (beta). Only in 2011 the categories of church membership added significantly to the model, as indicated by a significant beta-coefficient of 0.13, because Catholics were significantly more prejudiced than average, i.e., 0.23 points above the average of 1.89.^7^ Together, the characteristics in the model explained 24% of the individual variation in prejudice in 1985, while the explained variance dropped to 12% in 2011.

**Table 2:** Regression coefficients of educational attainment, church membership and church attendance on ethnic prejudice, expressed as the deviance from the sample mean in each survey year (N = 4780). *Source*: SOCON 1985–2011 (Eisinga et al. 1992, 1999, 2012)

	1985 (N = 1491)		1995 (N = 743)		2000 (N = 772)		2005 (N = 1029)		2011 (N = 745)	
	B	Beta^a^	B	Beta^a^	B	Beta^a^	B	Beta^a^	B	Beta^a^
Intercept	1.59***		1.72***		1.71***		2.03***		1.89***	
Educational attainment		.27***		.26***		.28***		.25***		.22***
Primary education	.38***		.30**		.32**		.43***		.52**	
Lower vocational education	.21***		.29***		.32***		.25***		.24**	
Lower secondary vocational	−.03		.03		.20*		.16*		.09	
Secondary vocational	.02		.05		.07		−.02		.02	
Upper secondary education	−.19**		−.16*		−.19*		.00		−.04	
Bachelor’s or equivalent level	−.34***		−.24***		−.29***		−.22***		−.13**	
Master’s or equivalent level	−.60***		−.28**		−.26**		−.39***		−.31***	
Church membership		.07		.09		.09		.05		.13**
Non-member	−.07		−.06		−.06		−.02		−.03	
Catholic church	.09		.10		.12		.08		.23**	
Protestant church	.01		.07		.05		−.03		−.12	
Church attendance		.02		.08		.04		.04		.09
No, hardly ever/never	.00		−.04		.00		−.02		−.04	
Yes, once or twice a year	.00		−.01		.04		.01		.06	
Yes, about once a month	.05		.17		−.06		.03		−.09	
Yes, about once a week	−.02		.05		−.06		.08		.16	
Variance explained	24%		21%		15%		16%		12%	

Effects are estimated simultaneously and controlled for cohort, socio-economic position, sex, urbanization, and province. The analyses are performed on the pooled data set after missing imputation, in which the results of the five imputations are combined

* *p* < .05; ** *p* < .01; *** *p* < .001 (two-tailed)

^a^To obtain a standardized coefficient which summarizes the effect of the dummy variables, we calculated a Sheaf-coefficient for each set of dummy variables (Heise 1972)

To analyze whether and how ethnic prejudice has changed over time within categories of education, church membership, and church attendance, we calculated the controlled estimated mean levels of prejudice for each category (i.e., by adding the intercept to the coefficients from Table 2), which are presented in Table 3. To test whether the mean level of prejudice in each category has changed significantly between two subsequent survey years, we used a statistical test for the difference between two regression coefficients across independent samples as suggested by Paternoster et al. (1998).

**Table 3:** Estimated mean levels of ethnic prejudice in each survey year per category of educational attainment, church membership and church attendance (intercept + coefficient) (N = 4780). *Source*: SOCON 1985–2011 (Eisinga et al. 1992, 1999, 2012)

	1985 (N = 1491)	1995 (N = 743)	2000 (N = 772)	2005 (N = 1029)	2011 (N = 745)	Change 1985–2011
Intercept	1.59	1.72*	1.71	1.88*	1.89	+0.30*
Educational attainment						
Primary education	1.97	2.03	2.03	2.31	2.40	+0.43*
Lower vocational education	1.80	2.01*	2.04	2.13	2.13	+0.33*
Lower secondary vocational	1.56	1.75	1.91	2.04	1.98	+0.42*
Secondary vocational	1.60	1.78*	1.78	1.86	1.90	+0.30*
Upper secondary education	1.40	1.56	1.53	1.88	1.84	+0.44*
Bachelor’s or equivalent level	1.25	1.48*	1.43	1.66*	1.76	+0.51*
Master’s or equivalent level	0.99	1.44*	1.45	1.49	1.58	+0.59*
Church membership						
Non-member	1.52	1.66*	1.66	1.86*	1.85	+0.33*
Catholic church	1.68	1.82	1.84	1.96	2.12	+0.43*
Protestant church	1.60	1.80*	1.76	1.85	1.77	+0.17
Church attendance						
No, hardly ever/never	1.59	1.68	1.71	1.86*	1.84	+0.26*
Yes, once or twice a year	1.59	1.72	1.76	1.88	1.94	+0.36*
Yes, about once a month	1.63	1.89*	1.65	1.91	1.79	+0.16
Yes, about once a week	1.57	1.77	1.65	1.96*	2.05	+0.48*

Controlled for cohort, socio-economic position, sex, urbanization, and province

* Significant mean difference compared to the previous survey year, tested with a Paternoster test (1998), *p* < .05 (two-tailed)

**Fig. 2:**
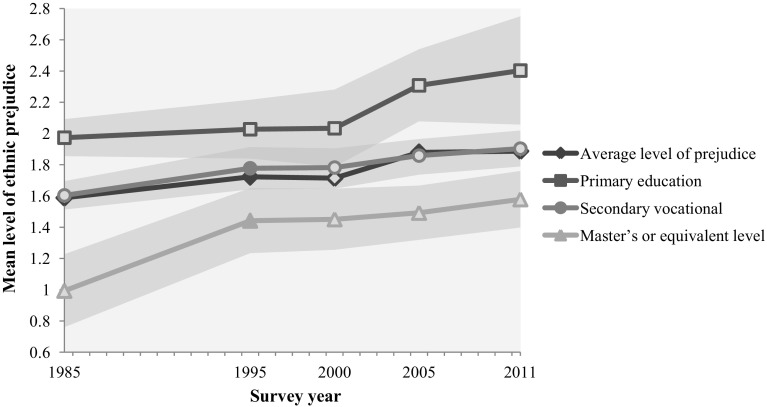
Controlled predicted mean level of ethnic prejudice and 95% confidence intervals for people with the lowest, middle and highest level of educational attainment, 1985–2011. *Note* Controlled for cohort, socio-economic position, sex, urbanization, and province. *Filled marker* indicates significant mean difference compared to previous survey year. *Grey areas* show the 95% confidence intervals. *Source*: SOCON 1985–2011 (Eisinga et al. 1992, 1999, 2012)

Table 3 shows that the trend in ethnic prejudice developed differently within each category of educational attainment. Between 1985 and 1995, particularly higher educated people became more prejudiced, as their mean level of prejudice rose significantly from 0.99 (1.59–0.60) to 1.44 (1.72–0.28). Consequently, higher educated individuals converged towards the lower and middle educated. The last column of Table 3 shows the increase in prejudice between 1985 and 2011, which was stronger among the higher educational levels and weaker among people with vocational training. The strongest increase is found among people with a master’s degree or equivalent level (+0.59, significant). This can also be seen in Fig. 2, in which we visualized the changes in ethnic prejudice among the lowest, middle and highest educated individuals.

Table 3 also shows significant increases in prejudice among Protestants, non-members and monthly churchgoers between 1985 and 1995, and among non-members and weekly churchgoers between 2000 and 2005. Yet, the last column of Table 3 shows that the overall increase in prejudice between 1985 and 2011 was strongest among Catholics (+0.43, significant) and weekly churchgoers (+0.48, significant), by which they diverged from the average prejudice level. Notwithstanding, the differences in prejudice levels between these groups remain very small.

### Counterfactual Analyses

**Fig. 3:**
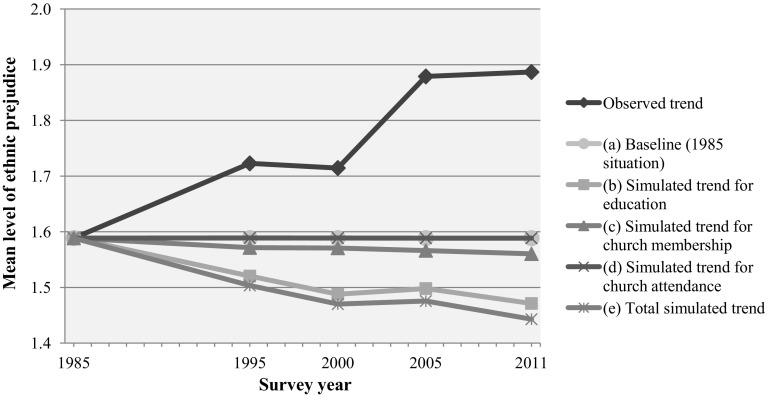
Observed and simulated trends in ethnic prejudice with differences between social categories on the 1985 level. *Source*: SOCON 1985–2011 (Eisinga et al. 1992, 1999, 2012)

**Fig. 4:**
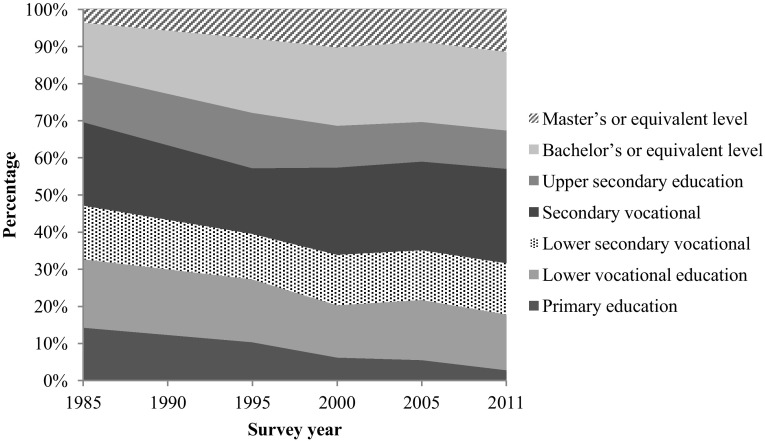
Changes in the educational distribution of the Dutch, 1985–2011. *Source*: SOCON 1985–2011 (Eisinga et al. 1992, 1999, 2012)

**Fig. 5:**
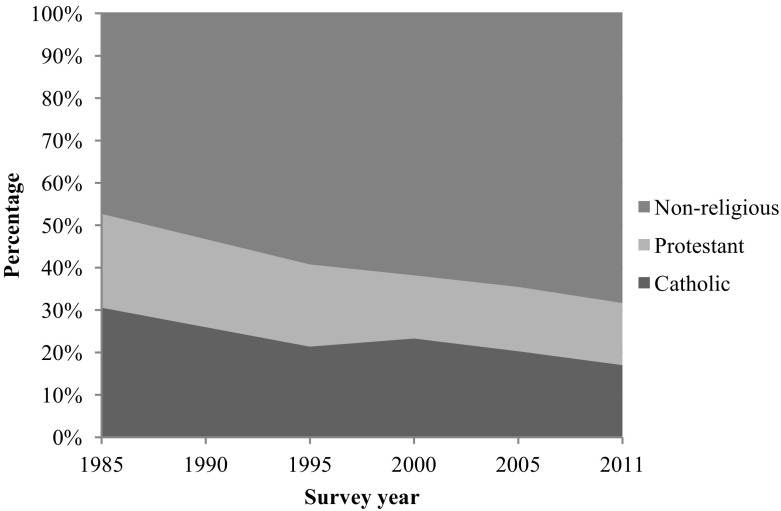
Changes in the distribution of church membership of the Dutch, 1985–2011. *Source*: SOCON 1985–2011 (Eisinga et al. 1992, 1999, 2012)

**Fig. 6:**
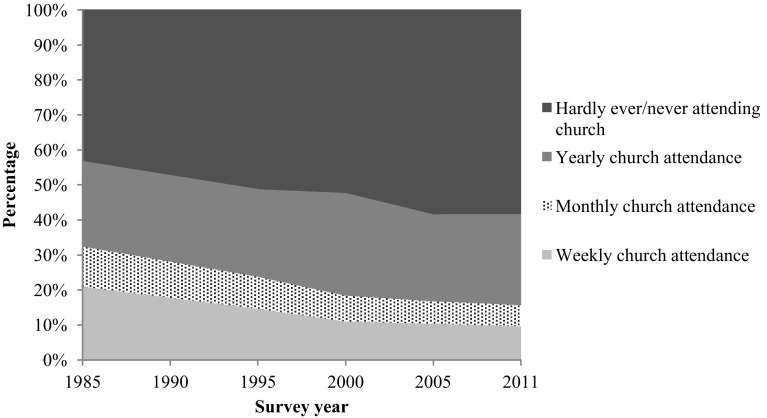
Changes in the distribution of church attendance of the Dutch, 1985–2011. *Source*: SOCON 1985–2011 (Eisinga et al. 1992, 1999, 2012)

To this point we have found a considerable change in levels of prejudice among the categories of educational attainment, while the changes within the categories of church membership and church attendance were limited (see Table 3; Fig. 2). Although prejudice on average rose significantly across all of these groups in society between 1985 and 2011, we found stronger increases among higher educated individuals, Catholics and weekly churchgoers. To test whether these differential changes have contributed significantly to the observed overall increase in prejudice, we used the outcomes of the multivariate regression analyses from Table 3 in a series of counterfactual simulations. Figure 3 graphically presents the observed trend in prejudice and the simulated trends resulting from simulations for which we held the levels of prejudice within each category of educational attainment, church membership and church attendance constant on the 1985 level, while taking into account the shifts in the distribution of these categories within the sample. The exact differences between the observed and simulated means and the corresponding bootstrapped confidence intervals are shown in Table 4.^8^

**Table 4:** Estimated differences between baseline/observed, religiosity/baseline and education/baseline and bootstrapped 95% confidence intervals (between square brackets). *Source*: SOCON 1985–2011 (Eisinga et al. 1992, 1999, 2012)

Year	1985	1995	2000	2005	2011
Baseline − observed (a)	0	−.13 [−.17, −.10]	−.13 [−.16, −.09]	−.29 [−.32, −.26]	−.30 [−.33, −.26]
Education − baseline (b)	0	−.07 [−.08, −.06]	−.10 [−.12, −.09]	−.09 [−.10, −.08]	−.12 [−.13, −.10]
Church membership − baseline (c)	0	−.02 [−.02, −.01]	−.02 [−.03, −.01]	−.02 [−.03, −.01]	−.03 [−.04, −.02]
Church attendance − baseline (d)	0	.00 ns [−.01, .01]	.00 ns [−.01, .01]	.00 ns [−.01, .01]	.00 ns [−.01, .01]
Total − baseline (e)^#^	0	−.09 [−.10, −.07]	−.12 [−.13, −.10]	−.11 [−.13, −.10]	−.15 [−.16, −.13]

*ns* non-significant with *α* = 0.05 two-tailed

^#^Row e is sum of ∆ education (b) + ∆ church membership (c) + ∆ church attendance (d)

In Fig. 3, the horizontal baseline (a) represents a simulated ‘null-model’ with no change in the levels of prejudice within categories of education, church membership, and church attendance, and no shift in the distribution of these characteristics since 1985. The exact differences between the flat baseline (no trend) and the actual observed trend are shown in Table 4, row (a). The corresponding confidence intervals all indicate that the observed means differ significantly from the baseline mean in each survey year. To illustrate, the difference between the 1985 baseline mean and the observed mean in 2011 is 0.30 (that is the difference between the intercepts 1.59 and 1.89 from Table 2). The corresponding bootstrapped 95% confidence interval for this difference indicates that the true difference in the population is to be found between 0.26 and 0.33.

Next, we simulated a new mean level of prejudice in each survey year for which only the levels of prejudice within education were held constant on the 1985 level, whereas all other levels as well as the sample distributions of all variables varied across subsequent years. Line (b) in Fig. 3 shows the resulting simulated trend. As previously demonstrated, the three highest educational levels showed a stronger increase in their mean levels of prejudice between 1985 and 1995 than the other educational categories (see Table 3; Fig. 2). Consequently, these categories converged towards the average level of prejudice (see Table 2). In Table 4, row (b) shows that if levels of prejudice within these educational categories had not changed since 1985, the overall level of prejudice would have significantly decreased with 0.07 points compared to the baseline which reflects the counterfactual situation in which everyone would have had the overall 1985 level of prejudice. Any difference between this baseline and line (b) reflects differential changes in levels of prejudice within the categories of education.

In addition, prejudice rose strongly among lower educated individuals between 2000 and 2011 (see Table 3; Fig. 2), who consequently moved further away from the average level of prejudice (see Table 2). Row (b) of Table 4 shows that the simulated trend continues to decline if these changes had not taken place and that this decline is significant in each survey year. To illustrate, the simulated mean prejudice level in 2011 would lie 0.12 points below the baseline. The corresponding confidence interval indicates that the true decline in the mean level of prejudice in the population probably lies somewhere between 0.10 and 0.13. Thus, the stronger rises among higher educated and lower educated people have both contributed to the observed trend in ethnic prejudice in the Netherlands, over and above the general rise in the average prejudice level. If these changes within the educational groups had not taken place, the rising proportion of higher educated people in the Netherlands would have resulted in a longitudinal decline in the mean level of prejudice in society. This supports hypothesis 1a (for period 1985–1995) and hypothesis 1b (for period 2000–2011).

Line (c) in Fig. 3 shows the simulated trend for a situation in which only the levels of prejudice within categories of *church membership* with prejudice were held constant on the 1985 level, whereas all other levels as well as all distributions could vary across the survey years. We found little influence of church membership on ethnic prejudice (see Table 2), consequently line (c) shows a minimal decline in the mean level of prejudice between 1985 and 2011. In row (c) of Table 4, for example, the simulated mean in 2011 would have been 0.03 points lower compared to the baseline. The corresponding confidence interval indicates that the true decline in the mean level of prejudice in the population probably lies somewhere between 0.02 and 0.04. Row (c) of Table 4 also shows that the differences between the baseline and the simulated means with constant levels for church membership in each survey year are significant, though very small. Thus, the rise in ethnic prejudice between 1985 and 2011 among Catholic church members (from 1.68 to 2.12, see Table 3), and to a lesser extent among non-members (from 1.52 to 1.85, Table 3) has contributed for a very small part to the observed trend, supporting both hypothesis 2a and 2c.

Line (d) in Fig. 3 demonstrates the simulated trend in prejudice for which we held only the prejudice levels within categories of *church attendance* constant on the 1985 level. In the previous section, we found little influence of church attendance on ethnic prejudice (see Table 2). As a consequence, the simulated trend (d), for which the rising shares of non-churchgoers are taken into account, hardly deviates from the baseline. This is confirmed by row (d) of Table 4, which shows no significant differences between the baseline means and the simulated means with constant levels for church attendance. Therefore, there is no support for hypothesis 2b and hypothesis 2d.

The separate contributions of differential changes in levels of ethnic prejudice within the various levels of educational attainment (b), church membership (c), and church attendance (d) add up to the total simulated trend (e) in Fig. 3. If these levels are all held constant since 1985, the average level of prejudice would have declined between 1985 and 2011. Table 4, row (e) shows that this decline amounts to 0.15 points below the baseline by 2011, and probably lies between 0.13 and 0.16 in the population. The confidence intervals in row (e) of Table 4 indicate that the difference between the total simulated means and the baseline is significant in each survey year.

## Conclusion and Discussion

This study was aimed at providing insights into the paradox of increasing shares of highly educated and non-religious individuals in Dutch society—categories which generally hold less prejudice—on the one hand, and yet, on the other hand, a longitudinal rise in ethnic prejudice in the Netherlands. Based on ethnic competition theory we formulated hypotheses on differential changes in prejudice within specific educational and religiously (non-)affiliated groups in Dutch society that could explain the observed rise in ethnic prejudice despite educational expansion and secularization. We added to previous research by testing the unique contribution of these differential changes to the observed trend in ethnic prejudice, while controlling for shifts in the distribution of these characteristics. For this purpose, we used five nationally representative cross-sectional surveys collected between 1985 and 2011 in counter factual analyses.

In line with prior studies, we found that lower educated people held significantly more, and higher educated people significantly less prejudice than average. While the average level of prejudice rose significantly between 1985 and 2011 across all educational categories in society, the increase was stronger among the higher educated, who thus converged towards the average level of prejudice, particularly between 1985 and 1995. This stronger increase in ethnic prejudice among higher educated individuals in particular could partially explain why an overall rise in ethnic prejudice is observed despite educational expansion. We further found a weak and largely non-significant influence of church membership and church attendance on prejudice. Although Catholics and weekly churchgoers showed a stronger increase in ethnic prejudice as compared to Protestants, non-church members, and people less frequently or never attending church, differences in prejudice between these groups remained largely absent. We showed that, as a consequence, rising shares of non-members and non-churchgoers have hardly resulted in a decline of prejudice. Additionally, *all* Dutch individuals have become more prejudiced between 1985 and 2011, irrespective of their educational level and religious affiliation, which has partly offset the supposed liberalizing influence of educational expansion and secularization. This development is not limited to the Netherlands. Research has shown similar increases in negative attitudes towards minorities, immigrants, and immigration in other European countries over the past decades (see Ceobanu and Escandell 2010), though studies into changes in ethnic prejudice are scarce.

This study has several implications for research on the relationship of education and religious affiliation with ethnic prejudice. Our results suggest that the liberalizing influence of education on ethnic prejudice as suggested by several researchers (e.g., Vogt 1997; Weil 1985) has decreased. Hence, the idea that liberal attitudes automatically diffuse from higher educated individuals to lower educated, in part through the educational system (Weil 1985), has contemporarily become open to doubt. Higher educated individuals do not seem immune to (perceptions of) ethnic threat, either due to increasing shares of ethnic minorities obtaining higher levels of education or due to a heterogenization of higher education, which may have changed the composition of the group higher educated individuals. Alternatively, some researchers have argued that higher educated are less prejudiced because they are more skilled at suppressing prejudiced responses in survey research and more sophisticated in defending their group ideology (Jackman and Muha 1984; Jackman 1978). Although this approach is sometimes contested, it provides an alternative explanation for our findings, namely that a taboo to express prejudice against ethnic minorities among higher educated individuals has been slowly disappearing. However, it is beyond the scope of our contribution to test the mechanisms behind the differential changes in ethnic prejudice among the educational categories. Therefore, we propose this as a direction for future research.

Moreover, we found the relationship of church membership and attendance with ethnic prejudice to be largely absent. This suggests that the generally accepted idea that religious affiliation strongly influences people’s level of ethnic prejudice may need refinement. This was also proposed by other researchers. For example, Hall et al. (2010) found that the positive relationship between extrinsic religiosity and racism declined over time in the United States. It seems that modernization has indeed eliminated the importance of religion (Norris and Inglehart 2011), although there might be a small group of religiously affiliated whose identity has become increasingly threatened in the secular Netherlands.

Finally, we showed that even substantial shifts in the relative shares of highly educated and secular individuals have had little impact on the general level of ethnic prejudice over time, because the differences in prejudice between higher and lower educated individuals declined and the differences between the religiously affiliated and non-affiliated were small to begin with.

Overall, these findings are perfectly in line with individualization theory (e.g., Beck and Beck-Gernsheim 2002; Felling et al. 2000), which proposes that people’s attitudes have become less and less determined by individual backgrounds and social institutions, such as their educational level and religious affiliation. Therefore, it is exactly this process of individualization that provides an answer to the paradox: as the importance of educational attainment and religious non-affiliation as barriers to ethnic prejudice have diminished, educational expansion and secularization have not resulted in the expected decrease in prejudice in the Netherlands over time.

Several limitations of this study should be acknowledged. Since data containing comparable measures of prejudice over such extended time periods are scarce, we could not determine whether the upward trend in prejudice is a recent or contemporary phenomenon, or had already started before 1985. In addition, educational expansion and secularization took off in the 1950s. Therefore, these processes may have actually reduced prejudice in the period before our data were collected. Likewise, we were not able to include more recent developments in ethnic prejudice. Although studies have shown slight decreases in negative public opinions towards the presence of ethnic minorities, immigration, and ethnic diversity in the Netherlands (Hjerm and Bohman 2014; Meuleman et al. 2009; van Setten et al. 2017), future research should indicate whether that also holds for ethnic prejudice. Moreover, we possibly underestimated the trend towards more prejudice because we were not able to examine prejudice against other ethnic minorities than the five groups in this research. Recently, the numbers of Eastern European and Muslim migrants have increased substantially in the Netherlands, which may have evoked stronger prejudice against these groups than against the five established minority groups included in the SOCON surveys. For example, Strabac and Listhaug (2008) found prejudice against Muslims in Europe to be more widespread than prejudice against other immigrants. In the Netherlands, a considerable share of recently migrated Poles and Bulgarians in the Netherlands reported perceptions of frequent discrimination of their own ethnic group and these perceptions have recently intensified (Gijsberts and Lubbers 2015; McGinnity and Gijsberts 2017).

Lastly, the question remains why ethnic prejudice has increased in the Netherlands ‘across-the-board’. Along with differential changes within particular social categories, it seems that *all* social categories have become somewhat more prejudiced over time, though some at a stronger pace than others. Certain societal circumstances could have affected all Dutch individuals similarly, further increasing the general level of ethnic prejudice. This supposition leads us to speculate on which societal circumstances may have reinforced the general level of prejudice. The persistent inflow of both economic and political migrants along with fluctuating numbers of refugees have repeatedly incited societal debates on the influx and presence of ethnic minorities. This might have increased perceptions of threat among all individuals in society. Moreover, from the 1980s onwards, several liberal Dutch politicians and opinion leaders (Bolkestein, Scheffer, Fortuyn, and more recently Wilders) have openly voiced concerns about immigration and poor integration of ethnic minorities. Ethnic minorities are increasingly framed as undermining the liberal Dutch values, which may have gradually legitimized the expression of prejudice against ethnic minorities in society, justified by an appeal to ‘free’ speech. Ironically then, the liberal values which have long been the basis of tolerance towards minorities have over time become a source of ‘free’ expressions of prejudice and exclusion. Unfortunately, we could not analyze which societal changes have contributed to the upward trend in prejudice due to the confounding of age, period and cohort explanations. Further research should address this question.

To summarize, a longitudinal increase in ethnic prejudice has taken place in the Netherlands despite educational expansion and secularization, which was the puzzling paradox we tried to solve. The answer is twofold. Firstly, the liberalizing influences of educational expansion and secularization have diminished over time: higher educated Dutch people have converged towards the secondary and lower educated Dutch, while the differences between the religiously affiliated and secular Dutch were largely absent from the beginning. Secondly, these processes have set all Dutch individuals ‘free’ to become more prejudiced over time. Because immigration of ethnic minorities into the Netherlands as well as to other European countries is not likely to cease, these findings suggest that the trend towards more ethnic prejudice will likely continue, heightening interethnic tensions in society.

## Appendix 1

See Table 5.

**Table 5:** Factor scores, eigenvalues and Cronbach’s alphas per survey year and in total. *Source*: SOCON 1985–2011 (Eisinga et al. 1992, 1999, 2012)

	1985 (N = 1606)	1995 (N = 800)	2000 (N = 831)	2005 (N = 1165)	2011 (N = 827)	Total (N = 5229)
With Moroccans you never know for certain whether they are going to be aggressive or not	.731	.607	.682	.651	.652	.715
Most people from Surinam work quite slowly	.691	.754	.640	.620	.591	.674
Gypsies are never to be trusted	.757	.713	.754	.650	.621	.668
Turks are backward	.602	.424	.586	.462	.594	.604
When you do business with Jews, you have to be extra careful	.706	.500	.631	.564	.609	.518
Eigenvalue	2.949	2.453	2.738	2.395	2.506	2.622
Cronbach’s alpha	.825	.735	.793	.727	.751	.772

## Appendix 2

See Figs. 4, 5 and 6.

## Appendix 3: Counterfactual Analysis: Simulating a Fixed Association

In this appendix, we explain counterfactual analysis in a straightforward example, using two categories of education and two survey years. In this example, 68.4% of the Dutch had a low or middle education and 31.6% had a high education in 1985 (see Table 6). Within the category of lower educated respondents, the percentage holding ethnic prejudice was 51.6% and among the higher educated it was 22.5%. In total, 42.2% of the sample were prejudiced.

**Table 6:** The association between education and ethnic prejudice in the Netherlands in 1985. *Source*: SOCON 1985–2011 (Eisinga et al. 1992, 1999, 2012)

Year: 1985	Low/middle education	High education	Row totals
No ethnic prejudice	419 48.4%	310 77.5%	729 57.6%
Ethnic prejudice	446 51.6%	90 22.5%	536 42.2%
Column totals	865 68.4%	400 31.6%	1265 100%

Strength of association in Cramér’s V = 0.27

In 2011, the educational distribution of our sample had changed considerably compared to 1985: the share of lower or middle educated Dutch decreased to 53.9% and the percentage higher educated increased to 46.1% (see Table 7). In total, the percentage holding ethnic prejudice increased with almost 20%, from 42.2% in 1985 to 60.8% in 2011. The relative share supporting ethnic prejudice increased to 68.5% (16.9% increase) among the low/middle educated respondents and to 51.7% (29.2% increase) among the higher educated respondents. The statistical association therefore dropped with 0.05 between 1985 and 2011 (Cramér’s V = 0.27 in 1985 and 0.22 in 2011).

**Table 7:** The association between education and ethnic prejudice in the Netherlands in 2011. *Source*: SOCON 1985–2011 (Eisinga et al. 1992, 1999, 2012)

Year: 2011	Low/middle education	High education	Row totals
No ethnic prejudice	119 31.5%	156 48.3%	275 39.2%
Ethnic prejudice	259 68.5%	167 51.7%	426 60.8%
Column totals	378 53.9%	323 46.1%	701 100%

Strength of association in Cramér’s V = 0.22

The observed increase in ethnic prejudice between 1985 and 2011 may be due to changes in the percentage prejudiced individuals within the categories of higher and lower educated respondents (i.e. changes in the association between education and prejudice). Previous research has shown that higher educated individuals express less prejudice than the lower educated, which is supported by the percentages in Tables 6 and 7. To analyze the magnitude of changes in prejudice within the two educational categories, we simulated a counterfactual situation for which we used the 1985 association together with the 2011 distribution of education and the 2011 sample (see Table 8).

**Table 8:** The simulated association between education and ethnic prejudice in the Netherlands, with the 1985 association and the 2011 distribution of education and sample size. *Source*: SOCON 1985–2011 (Eisinga et al. 1992, 1999, 2012)

Year: 1985	Low/middle education	High education	Row totals
No ethnic prejudice	183 48.4%	250 77.5%	433 61.8%
Ethnic prejudice	195 51.6%	73 22.5%	268 38.2%
Column totals	378 53.9%	323 46.1%	701 100%

In Table 8 the column total percentages show the sample size and the distribution of the low/middle educated and the highly educated in 2011. The inner cell percentages on which the association is based are from 1985. As was shown in Table 6, 51.6% of the low/middle educated and 22.5% of the highly educated were prejudiced in 1985. With these percentages (from 1985) and the absolute numbers in the column totals (from 2011) we can calculate the new absolute numbers in the inner cells. There are 195 respondents with prejudiced attitudes among the low/middle educated (.516*378) and only 73 among the higher educated (.225*323). Finally, we summed both absolute numbers and calculated the simulated percentage of prejudiced respondents, which amounted to 38.2% ((195 + 73)/701). Thus, if the association between education and prejudice had not changed since 1985, then the percentage prejudiced individuals in 2011 would have been 38.2 and not 60.8. This means that the shift in the percentage of higher educated and low/middle educated individuals with ethnic prejudice is associated with a decline in prejudice of 22% points (60.8–38.2).

## Footnotes

1 Factor analyses performed before and after imputation yielded similar results.

2 Factor scores were preferred to Likert scores, as factor scores take account of the factor structure of the data and different weights are applied to items according to the factor loadings. The trend in ethnic prejudice is highly comparable between factor scores and Likert scores.

3 We controlled for the province respondents live in, because levels of prejudice differ considerably across the twelve Dutch provinces.

4 In the survey of 1985 a different categorisation was used which was not comparable with the address density measure of the other waves. Therefore, we classified the municipalities of the 1985 survey according to their score on degree of urbanisation in 1995. These data are retrieved from Statistics Netherlands (Statistics Netherlands 1995).

5 Separate investigation of the five items revealed that three items showed a similar pattern, i.e., prejudice against Moroccans, Surinamese and gypsies has increased over the years, whereas two items deviated slightly from this pattern. To test for robustness of the findings, the multivariate regression model was tested on a scale based on these three items. The results did not deviate notably from the model including all five minority groups. As an additional robustness test, we estimated a model without Jews and Gypsies. The results are virtually similar to the model with all five minority groups included. We also estimated the model for Turks and Moroccans only, as it is argued that prejudice against Muslims in particular might have increased. Again, there are no substantial differences between this model and a model including all five minority groups.

6 The overall increase in prejudice is significant (F = 23.11, p < 0.001). A post hoc Bonferroni test revealed that the change in the mean between 1995 and 2000 and between 2005 and 2011 was not significant at the 0.05 level (two-sided).

7 Additional regression analyses (not shown here) indicated that the non-significant difference in prejudice between the categories of church attendance is partly due to the inclusion of church membership.

8 The sampling distribution of the differences between the observed and the simulated means is unknown and depends on the estimated and simulated effects, on the observed distributions, and the co-variances between all variables. Therefore, we used a bootstrapping procedure to empirically determine the shape of the sample distribution for each estimated difference in Table 4. For each survey year, we drew 25,000 bootstrap samples (5,000 for each of the 5 imputation data sets) with replacement from the original sample. Based on these samples we could determine the lower and upper boundaries of the 95% confidence interval to test the significance of the difference between the observed means and our simulated means. For instance, the difference between the total simulated mean in 2011 and the baseline mean (the observed mean in 1985), which amounts to -0.15 will most likely lie somewhere between -0.16 and -0.13 in the target population (see Table 4, row (e)).
